# Unraveling the Role of Astrocytes in Subthalamic Nucleus Deep Brain Stimulation in a Parkinson’s Disease Rat Model

**DOI:** 10.1007/s10571-019-00784-3

**Published:** 2020-01-14

**Authors:** Ana Carolina Pinheiro Campos, Daniel Seicho Kikuchi, Amanda Faure Nardini Paschoa, Mayra Akemi Kuroki, Erich Talamoni Fonoff, Clement Hamani, Rosana Lima Pagano, Marina Sorrentino Hernandes

**Affiliations:** 1 Division of Neuroscience, Hospital Sírio-Libanês, São Paulo, SP 01308-060 Brazil; 2 Department of Medicine, Emory University, Atlanta, GA 30322 USA; 3 Division of Neurosurgery, Department of Neurology, University of São Paulo Medical School, São Paulo, 01246-903 Brazil; 4 Sunnybrook Health Research Institute, Harquail Centre for Neuromodulation, Toronto, ON M4N 3M5 Canada

**Keywords:** Deep brain stimulation, High-frequency stimulation, Parkinson’s disease, Inflammation, Astrocytes, NF-κB

## Abstract

Deep brain stimulation (DBS) of the subthalamic nucleus (STN) is an effective therapeutic strategy for motor symptoms of Parkinson’s disease (PD) when L-DOPA therapy induces disabling side effects. Classical inflammatory activation of glial cells is well established in PD, contributing to the progressive neurodegenerative state; however, the role of DBS in regulating the inflammatory response remains largely unknown. To understand the involvement of astrocytes in the mechanisms of action of DBS, we evaluated the effect of STN–DBS in regulating motor symptoms, astrocyte reactivity, and cytokine expression in a 6-OHDA-induced PD rat model. To mimic in vivo DBS, we investigate the effect of high-frequency stimulation (HFS) in cultured astrocytes regulating cytokine induction and NF-κB activation. We found that STN-DBS improved motor impairment, induced astrocytic hyperplasia, and reversed increased IFN-γ and IL-10 levels in the globus pallidus (GP) of lesioned rats. Moreover, HFS activated astrocytes and prevented TNF-α-induced increase of monocyte chemoattractant protein-1 (MCP-1) and NF-κB activation in vitro. Our results indicate that DBS/HFS may act as a regulator of the inflammatory response in PD states, attenuating classical activation of astrocytes and cytokine induction, potentially through its ability to regulate NF-κB activation. These findings may help us understand the role of astrocyte signaling in HFS, highlighting its possible relationship with the effectiveness of DBS in neurodegenerative disorders.

## Introduction

Deep brain stimulation (DBS) has been used worldwide in clinical practice with excellent results in several neurological diseases, including movement disorders, especially Parkinson’s disease (PD) (Benabid 2003). Surgical candidates are those who either became refractory to conventional treatments or developed serious drug-induced side effects. DBS, as used to treat PD patients, involves applying high-frequency stimulation (HFS) through electrodes implanted in strategic nuclei, with the ability to adjust stimulation parameters to achieve optimal therapeutic benefits (Cicchetti and Barker 2014). Subthalamic nucleus (STN) DBS leads to improvements in motor symptoms, including rigidity, tremor, and bradykinesia, as well as L-DOPA-induced dyskinesias (Krack et al. 1997; Hamani et al. 2004; Goodman et al. 2006), improving patient's quality of life (Lagrange et al. 2002; Diamond and Jankovic 2005). Despite the widespread effectiveness of this technique, the specific mechanisms though which DBS improves clinical symptoms are still unclear. Data generated so far supports a role for DBS in modulating neurotransmission by altering the firing pattern of neurons (Beurrier et al. 2001; Dostrovsky and Lozano 2002; Hamani et al. 2011; Chiken and Nambu 2016). However, the output response of the STN after DBS is still unclear and contradictory (Benazzouz et al. 2000; Hamani et al. 2004, 2017; Harnack and Kupsch 2010; Hashimoto et al. 2013; Florence et al. 2016). While DBS does not seem to prevent dopaminergic degeneration in PD patients (Piboolnurak et al. 2007), in preclinical experiment stimulation, it has been shown to protect against dopaminergic loss in the nigrostriatal pathway (Maesawa et al. 2004; Spieles-Engemann et al. 2010).

The importance of glial response in the neurodegenerative process of PD has been previously described (McGeer and McGeer 2008; Stott and Barker 2014). In response to a brain insult, reactive glial cells, such as astrocyte and microglia, release cytokines and reactive oxygen species (Dauer and Przedborski 2003; Sofroniew and Vinters 2010), which may contribute to neuronal cell damage and neurodegeneration (Anglade et al. 1997; Obeso et al. 2004). The transcriptional regulation of cytokines is mediated by the activation of the nuclear factor kappa-light-chain-enhancer of activated B cells (NF-κB). The primary mechanism for canonical NF-κB activation includes the inducible degradation of the nuclear factor of kappa light polypeptide gene enhancer in B-cell inhibitor (IκB)-α (Duh et al. 1989; Verma et al. 1995; Gupta et al. 2010).

Reactive astrocytes can undergo a phenotypic switch from classical inflammatory (A1) to alternative anti-inflammatory (A2), in analogy to the M1/M2 polarization for microglia/macrophage (Liddelow et al. 2017). It has been widely recognized that astrocytes adopt neurotoxic or neuroprotective phenotypes depending on the nature of the immune or inflammatory microenvironment (Jang et al. 2013; Jha et al. 2015). Thus, the inflammation-mediated neurodegenerative response depends not only on the presence of reactive astrocytes at the injury site, but also on their A1 or A2 functional polarization. Astrocytes are essential components of chemical synapses integrating the tripartite synapse (Araque et al. 1999; Perea et al. 2009). By releasing and uptaking neurotransmitters such as GABA, glycine, and glutamate, astrocytes are able to optimize and modulate neuronal communication (Sofroniew and Vinters 2010). However, in their A1 polarization state, the inflammatory phenotype inhibits the glutamate reuptake transporters altering the formation and quality of new synapses (Korn et al. 2005; Liddelow et al. 2017); hence, controlling inflammation is essential to regulate the synapses disruption. The role of DBS in reducing hippocampal neuroinflammation in epileptic rats has been previously described (Amorim et al. 2015), and the importance of astrocytes in response to stimulation has been discussed (Fenoy et al. 2014), but not yet tested. We postulated that DBS/HFS modulates classical inflammatory activation of astrocytes, contributing to the overall control of neuroinflammation in PD. To test this hypothesis, we characterized the effects of STN–DBS on the astrocytic phenotype of the globus pallidus (GP), an output nucleus of the STN, as well as on motor symptoms induced by 6-hydroxydopamine (6-OHDA) in a rat model of PD. In addition, to further understand the effects of DBS in astrocytes, we delivered HSF to cultured astrocytes prior to stimulation with tumor necrosis factor (TNF)-α, which has been found to be increased in PD (Boka et al. 1994; Mogi et al. 2000; Nagatsu and Sawada 2005; Sawada et al. 2006). Our findings uncover a previously unknown role of HFS/DBS in astrocyte activation and advance our understanding of the mechanism involved in DBS in brain areas surrounding the stimulation target.

## Materials and Methods

### Experimental Animals

A total of 40 male Wistar rats (200–250 g) were used in this study. Rats were housed in acrylic boxes (3 rats per box) for at least a week before the experimental procedures were initiated. The animals were maintained in appropriate rooms with controlled light/dark cycle (12/12 h) and temperature (22 ± 2 °C) with wood shavings and free access to water and rat chow pellets. All animal experiments were conducted and reported in accordance with the ARRIVE guidelines (https://www.nc3rs.org.uk/arrive-guidelines). The protocols used during the execution of this project were approved by the Ethics Committee on the Use of Animals (CEUA) at Hospital Sírio-Libanês (SP, BRA), under protocol number CEUA 2016/04.

## Surgical Procedures for PD Model Induction and Electrode Implantation

Animals were anesthetized with isoflurane (4% induction, 2.5% maintenance in 100% oxygen) associated with local anesthesia (2% lidocaine, 100 μL/animal on the scalp). Twelve µg of 6-hydroxydopamine (6-OHDA, Sigma-Aldrich, MO, USA) diluted in 2 μL of 0.9% saline with 0.2% ascorbic acid was injected into two different points of the left striatum (6 μg/µL of 6-OHDA in each point) (Chudler and Lu 2008) under stereotaxic conditions. The injections were performed using a Hamilton syringe at the following coordinates: + 2.7 mm mediolateral, 0.0 mm anteroposterior, and + 4.5 mm dorsoventral (first point); + 3.2 mm mediolateral, + 0.5 mm anteroposterior, and + 4.5 mm dorsoventral (second point), according to the rat brain atlas (Paxinos and Watson 2005). Control groups included animals injected with 1 μL of saline in two different points of the left striatum. At the end of the injection, the needle was held in place for an additional 5 min to prevent backflow of the solution. In addition to 6-OHDA injections, a separate group of animals was implanted with insulated stainless steel electrodes (250 μm in diameter with 0.55 mm of surface exposed, Plastic One, CA, USA) into the left STN during the same surgical procedure. These electrodes were used as cathodes and implanted in the following coordinates: + 2.5 mm mediolateral, − 3.7 mm anteroposterior, and + 7.5 mm dorsoventral (Paxinos and Watson 2005). Screws implanted on the skull over the midbrain (− 6.0 anteroposterior and + 2.5 lateral) were used as anodes (Paxinos and Watson 2005). Electrodes were fixed to the skull with dental acrylic cement. After the striatal injection and implantation of the electrode, animals were treated with meloxicam®, a non-steroidal anti-inflammatory drug (NSAID) (0.5 mg/kg, SQ, Ourofino Pet, SP, BRA) and penicillin/streptomycin as prophylactic antibiotics (0.2 mg/kg, i.p., Zoetis, SP, BRA). Rats were returned to their home cages and monitored until complete recovery from anesthesia. The regular diet was supplemented with a dietary supplement (Ensure, Abbott, SP, BRA) once a day for 3 consecutive days to ensure full recovery of the animals after the nigrostriatal injury. Following euthanasia, the location of the implants was confirmed through the Nissl-stained section.

## DBS Protocol

Seven days after PD model induction and electrode implantation, a group of 6-OHDA animals were treated with 5 sessions of DBS (6-OHDA + DBS ON—biphasic cathodic pulses at 130 Hz, 60 µsec pulse width, 0.1 mA, 2 h/day) using a portable stimulator (St Jude MTS, St Jude Medical, Plano, TX, USA). DBS was applied for 5 days from 9:00 AM to 11:00 AM. Control animals received 6-OHDA injections and had electrodes implanted, but no stimulation was delivered (6-OHDA + DBS OFF).

## Experimental Design—In Vivo

**Fig. 1:**
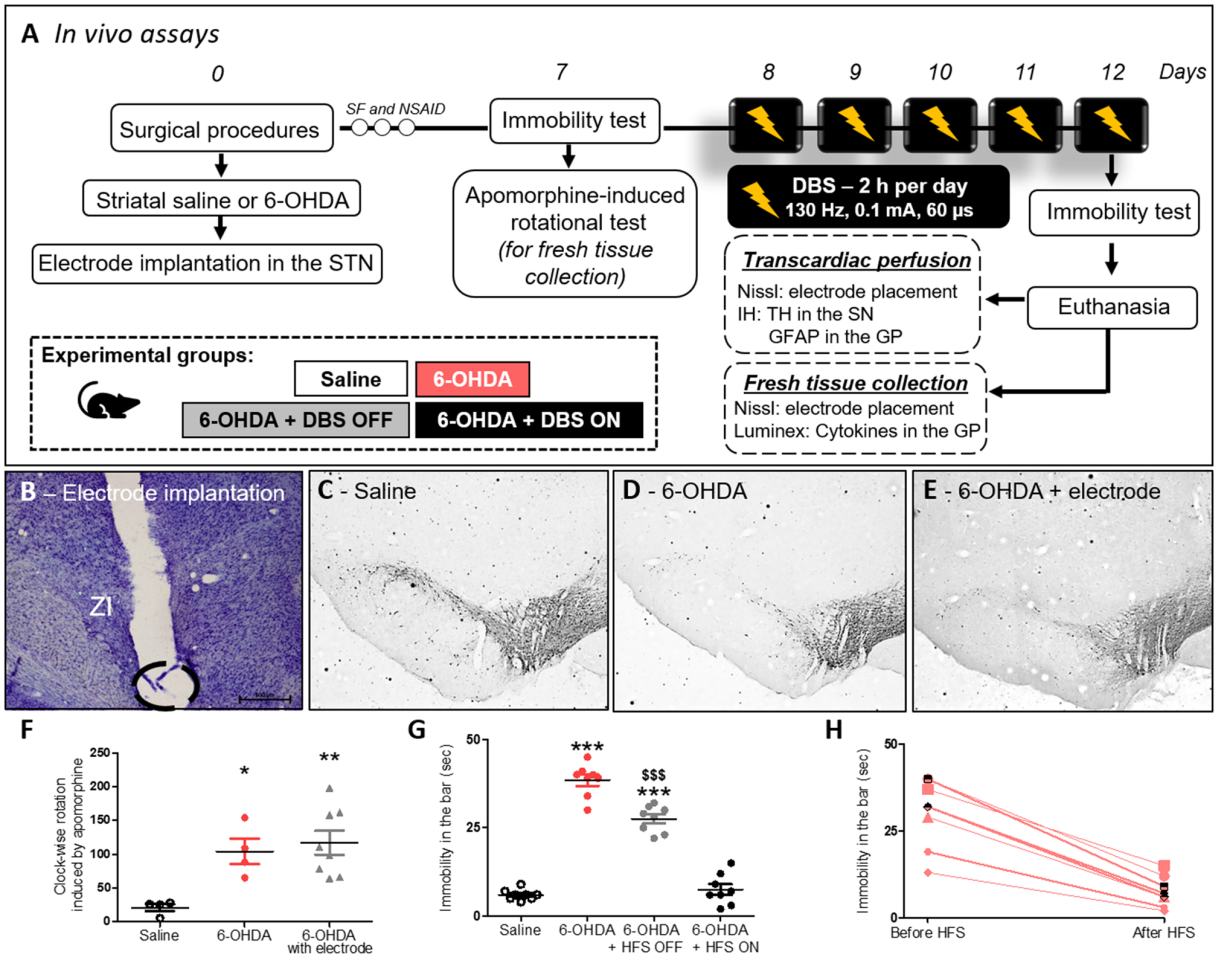
Effect of STN-DBS on apomorphine-induced rotational behavior and immobility induced by 6-OHDA. **a** In vivo experimental design. **b** Nissl staining analysis to confirm electrode implantation in the STN. Representation of 6-OHDA-induced PD model in saline, 6-OHDA, and 6-OHDA + electrode OFF groups. **c**–**e** Immunohistochemistry for TH in the striatum showing 6-OHDA-induced dopaminergic degeneration in the SN. Images are representative of four independent experiments. **f** Apomorphine-induced rotational behavior was evaluated in order to confirm the dopaminergic deficit. Values represent the mean + SEM (*n* = 4–8). *p < 0.05; **p < 0.001 vs. control saline group. **g** In vivo evaluation of immobility test following STN–DBS. Values represent the mean + SEM (*n* = 8 per group). ***p < 0.0001 vs. control saline group and $$$*p* < 0.0001 vs. 6-OHDA group. **h** Comparison between the immobility test performance before and after STN–DBS (immobility in the bar vs. before HFS in seconds) (*n* = 8 per group)

Forty rats were randomly assigned to receive striatal 6-OHDA or saline injections under stereotaxic conditions, as described above. During the same surgical procedure, a separate group of animals had electrodes implanted into the left STN. Experimental groups were divided as follows: (1) Animals injected with striatal saline (*n* = 8), (2) Animals injected with striatal 6-OHDA (*n* = 8), (3) Animals injected with striatal 6-OHDA + DBS OFF (only electrode implanted) (*n* = 12), and (4) Animals injected with striatal 6-OHDA + DBS ON (stimulated) (*n* = 12). Seven days after the surgical procedure, animals were evaluated using immobility and rotation behavioral tests. From the 8th to the 12th day after the surgical procedure, 6-OHDA + DBS ON rats received DBS, as described above. After the last stimulation session (12 days after the surgical procedure), all experimental groups were re-evaluated in the immobility test. Immediately after the last behavioral test, half of the animals underwent transcardiac perfusion and their brains were collected to verify the correct positioning of the STN–DBS electrodes and for the evaluation of immunoreactivity (IR) for tyrosine hydroxylase (TH) in the substantia nigra (SN) and glial fibrillary acidic protein (GFAP) in the GP. Additionally, the other half of the animals were randomly selected and fresh brain tissue containing GP was quickly collected. Both electrode placement and cytokine levels (interleukin (IL)-1β, IL-6, IL-10, and interferon (IFN)-γ) were evaluated (Fig. 1a).

## Apomorphine-Induced Rotational Behavior

Apomorphine-induced rotational behavior was evaluated in order to validate the PD model, since the number of asymmetric rotations correlates with the degree of nigral degeneration, as previously demonstrated (Domenici et al. 2019). Rotational asymmetric behavior was evaluated using an automatic rotometer system (Rota-Count 8, Columbus Instruments, OH, USA) 7 days after striatal saline or 6-OHDA injections. Animals receiving saline, 6-OHDA, 6-OHDA + DBS OFF, and 6-OHDA + DBS ON were injected with the dopaminergic agonist, apomorphine (1 mg/Kg, s.c., Tocris Bioscience, BZ, UK), dissolved in 0.9% saline, and evaluated over 30 min, as previously described (Zhang et al. 2008). The criterion for rotation was a 180° turn toward the side contralateral to the lesion. To reduce stress, rats were exposed to the automatic rotometer system for 30 min one day before the rotational test. None of the animals injected with 6-OHDA failed to present asymmetric rotational behavior and therefore no animals were excluded from the study. Rats injected with saline in striatum were also evaluated.

## Behavioral Immobility

Seven days after PD model induction and following the fifth DBS session (day 12), animals were evaluated in the bar test to measure akinesia (typical catalepsy test). Saline control animals and 6-OHDA-injected animals that were not subjected to DBS were also evaluated after 12 days of striatal injections. The immobility test consists of placing an animal in an unusual posture and recording the time that the animal takes to correct his posture (Sanberg 1980). Behavioral immobility was characterized by muscle rigidity and failure to correct an imposed posture for a prolonged period. In this test, the animals were positioned with both forepaws on a 9-cm horizontal bar (0.9 cm diameter). The time course during which the animal remained motionless in this imposed posture was considered the bar test elapsed time (with a cutoff time of 120 s). The behavioral immobility endpoint was considered when both forepaws were removed from the bar or when the animal moved its head in an exploratory manner.

## Immunohistochemistry

After the last behavioral test (12 days), animals were anesthetized with ketamine/xylazine (0.5/2.3 mg/Kg, respectively, i.p.) and then submitted to transcardial perfusion with 0.9% saline solution, followed by 4% paraformaldehyde (PFA) dissolved in 0.1 M phosphate buffer (PB, pH 7.4). Their brains were collected and postfixed in PFA for 4 h, followed by incubation with 30% sucrose solution in PB for 48 h at 4 °C. Tissue sections (40 μm), obtained using a freezing sliding microtome, were kept under constant agitation and subjected to the following steps: (a) Incubation for 12–16 h at 4 °C with specific primary antibodies to mouse anti-TH (1:1000, MAB5280, Millipore, MA, USA) or mouse anti-GFAP (1:1000, G3893, Sigma-Aldrich) diluted in 0.3% triton X-100, containing 5% normal donkey serum (Jackson ImmunoResearch, ME, USA); (b) Incubation for 2 h at room temperature with biotinylated secondary antibodies (1:200, Jackson ImmunoResearch), and (c) Incubation with avidin–biotin-peroxidase complex (1:100, ABC Elite kit, Vector Labs, CA, USA). Labeling was developed with 0.05% diaminobenzidine tetrahydrochloride (DAB, Sigma-Aldrich) and 0.03% hydrogen peroxide in PB. Tissue sections were washed between each step (3 × 10 min). The sections were mounted on glass slides, air-dried, dehydrated, and coverslipped. Finally, images were obtained utilizing a light microscope (Eclipse E1000, Nikon, NY, USA). The regions of interest, including the SN (from bregma − 6.60 to − 6.00 mm anteroposterior, − 1.00 to − 3.2 mm mediolateral, and − 7.2 to − 8.8 mm dorsoventral) and GP (from bregma − 1.44 to 3.72 mm anteroposterior, − 2.4 to − 3.8 mm mediolateral ,and − 5.00 to − 7.8 mm dorsoventral), were identified based on a stereotaxic atlas (Paxinos and Watson 2005). Using ImageJ software (National Institutes of Health, MD, USA; https://rsbweb.nih.gov/ij/), the TH–IR and the number of GFAP-positive cells were analyzed per area (mm^2^). Measurements were taken from five sections per animal and five animals per group.

## Luminex

After the last behavioral test (12 days), half of the animals were euthanized by decapitation, the GP was freshly dissected and gently homogenized at 4 °C in radioimmunoprecipitation assay (RIPA) buffer (50 mM Tris, 150 mM NaCl, 1 mM EDTA, 0.1% SDS, 0.5% deoxycholate, 1% NP-40) with fresh protease inhibitors. The Luminex assay was used to quantify levels of IL-1β, IL-6, IL-10, and IFN-γ (RECYTMAG-65 K, Millipore). The assay was carried out in accordance with the manufacturer’s recommendations.

## Nissl Staining

The confirmation of electrode placement in the STN was evaluated by a retrospective analysis of Nissl-stained coronal sections. In half of the animals in each group, the fixed brain sections obtained using a freezing sliding microtome, as described above, were mounted onto gelatin-subbed slides. In the other half of the animals, slices approximately 2 cm thick containing the GP were freshly collected and frozen in OCT compound (Sigma-Aldrich). Tissue sections (20 μm) were obtained using a cryostat and mounted onto gelatin-subbed slides. Brain sections were then incubated in cresyl violet solution (1 g of cresyl violet + 10 mL of 100% acetic acid and 1 L of distilled water) for 30 min at room temperature, washed in distilled water, dehydrated in an ascending ethanol series (70%, 95%, and 100%), and incubated in xylene solution. Sections were mounted in Permount Mounting Medium and the images were acquired using a light microscope (Eclipse E1000, Nikon).

## Cell Culture and HFS Stimulation

C8-D1A mouse type I astrocytes (CRL-2541, ATTC, MD, USA) were cultured in DMEM/F12 media (Invitrogen, MA, USA) supplemented with 10% fetal bovine serum (FBS), 2 mM/L L-glutamine, 100 units/mL penicillin, and 100 mg/mL streptomycin. Cells were plated on collagen-coated culture dishes and the medium was changed every 2 days until cells reached 80% confluence. Cells were used between passages 4 to 10 and seeded at a density of 2 × 10^5^ cells per 60 mm^2^ dish for experiments.

## Experimental Design—In Vitro

TNF-α was used to stimulate astrocytes mimicking higher concentrations found in response to induced PD model (Boka et al. 1994; Mogi et al. 2000; Nagatsu and Sawada 2005). Cells were incubated in 0.1% FBS in DMEM/F12 media overnight and stimulated with TNF-α (100 ng/mL, PeproTech, NJ, USA) for 1, 3, 6 or 24 h (Fig. 3a). IL-6 and monocyte chemoattractant protein (MCP)-1 mRNA levels were analyzed. In each experiment, cultures exposed to TNF-α were compared with PBS control conditions. In order to analyze NFκ-B activation, IκB-α degradation was investigated at different time points following TNF-α stimulation for 15, 30, or 60 min (Fig. 3b). For the investigation of HFS, cells were cultured in plastic dishes and two monopolar tungsten electrodes were submerged in media with care taken to ensure no direct contact with the cell layer. The electrodes were connected to the same portable stimulator (St Jude MTS) used for in vivo experiments. Cultured astrocytes were stimulated with biphasic cathodic pulses at 130 Hz (0.1 mA and 60 μsec pulse width) for 6 h through. Cells were subjected to HFS for 6 h and during the last hour of stimulation TNF-α was added to the culture media. IL-6 and MCP-1 levels were analyzed at the mRNA and protein levels (Fig. 4a). Additionally, cells were subjected to HFS for 6 h and during the last 15 min of stimulation TNF-α was added to the culture media and NFκ-B activation was evaluated. Both IκB-α degradation (in whole cell lysate) and p65 nuclear translocation (in subcellular fractionation) were analyzed. Additional control conditions included cells with or without TNF-α and not electrically stimulated (Fig. 5a).

## Real‑Time Polymerase Chain Reaction (RT-PCR)

Total RNA was extracted with the RNeasy Plus kit (Qiagen, MA, USA). Reverse transcription was performed using Superscript II reverse transcriptase (Invitrogen) with random primers and cDNA was purified with the QIAquick kit (Qiagen). cDNA was amplified with primers against IL-6 (F:GTCTATACCACTTCACAAGTC,R:TGCATCATCGTTGTTCATAC), MCP-1 (F:AGCACCAGCCAACTCTCACT, R:TCTGGACCCATTCCTTCTTG), and RPL (housekeeping gene—F:ATGACAAGAAAAAGCGGATG, R:CTTTTCTGCCTGTTTCCGTA) using Platinum Taq DNA polymerase (Invitrogen,) in the presence of SYBR green I. Reactions were carried out in glass capillaries, using the LightCycler 1.2 (Roche, MA, USA) real time thermocycler. Data analysis was performed using the mak3 module of the qpcR software library in the R environment.

## Western Blotting

### Whole Cell Lysate

Whole cell lysate was prepared using triton buffer (25 mM HEPES, 100 mM NaCl, 1 mM EDTA, and 1% Triton x-100) with 10 μg/mL aprotinin, 10 μg/mL leupeptin, 1 mM PMSF, and Halt phosphatase inhibitor cocktail (78,428, Thermo Fisher Scientific, CA, USA). Samples were processed using a tissue homogenizer before sonification and centrifugation. The Bradford assay (Bio-Rad, CA, USA) was used to measure protein concentrations. The samples were diluted in Laemmli buffer for separation using SDS-PAGE. Following electrophoretic separation, proteins were transferred to a PVDF membrane (0.2 μm in diameter, Millipore), blocked for 1 h at room temperature with 5% BSA in Tris-Saline buffer, and the membranes were incubated overnight at 4 °C with the rabbit anti-IκB-α (1:2000, #ab32518, Abcam, UK) or rabbit anti-β-tubulin (1:5000, #ab6046, Abcam) diluted in 0.1% Tween-20 (TBST). The membranes were then washed with TBST and incubated for 2 h with the appropriate peroxidase-labeled secondary antibodies (1:2000, Amersham Biosciences, NJ, USA) diluted in TBST. The excess conjugate was removed with an added further wash cycle and the antigens were developed using the chemiluminescence ECL Kit (Amersham Biosciences) and analyzed for the density of the labeled bands using the ImageJ software. The anti-β-tubulin was used as loading control and the control group was normalized to 100 for comparison with other groups.

## Subcellular Fractionation

Whole cell lysate was prepared from cultured astrocytes using NP-40 buffer (0.1% NP40 with PBS). Homogenates were centrifuged at 10,000 rpm. The supernatant (cytosolic fraction) was collected and the nuclear pellet was resuspended in NP-40 buffer and further pelleted. The final nuclear pellet was then resuspended in 0.1% Triton and Laemmli buffer (Bio-Rad) for separation by SDS-PAGE, as described above. The membranes were incubated overnight at 4 °C with the rabbit anti-p65 (1:1000, #8242, Cell Signaling, MA, USA), rabbit anti-Histone 3 (1:500, #07–354, Millipore), or rabbit anti-β-tubulin (1:5000, Millipore) diluted in TBST. The membranes were then washed with TBST and incubated for 2 h with the appropriate peroxidase-labeled secondary antibodies (1:2000, Amersham Biosciences) diluted in TBST. The excess conjugate was removed with a further wash cycle and the antigens were developed using the chemiluminescence ECL Kit (Amersham Biosciences) and analyzed for the density of the labeled bands using the ImageJ software. Histone 3 and β-tubulin were used as loading controls for nuclear and cytosolic fractions.

## ELISA

Whole cell lysate was prepared from cultured astrocytes using a radioimmunoprecipitation assay (RIPA) buffer (50 mM Tris, 150 mM NaCl, 1 mM EDTA, 0.1% SDS, 0.5% deoxycholate, and 1% NP-40) with fresh protease inhibitors. MCP-1 expression analysis in cell lysate and cell supernatant was performed according to the manufacturer’s instructions (R&D, Minneapolis, MN, USA).

### Statistical Analysis

For animal studies, results obtained were expressed as means ± standard error of the mean (SEM). For cell culture results, data are expressed as means ± standard deviation of the mean (SD). Data were analyzed using GraphPad Prism (CA, USA) and statistical significance was assessed using ANOVA, followed by Tukey’s multiple comparison post hoc tests. Analysis of akinesia before and after DBS was calculated using the paired *t*-test. In all cases, *p* < 0.05 was considered statistically significant.

## Results

### STN-DBS Improves Motor Impairment

Rats subjected to a unilateral 6-OHDA-induced PD model presented loss of TH-IR in the SN pars compacta (Fig. 1d and e) and asymmetric rotation to the contralateral side of the lesion (1-w-ANOVA; *F*_(2,15)_ = 7.524, *p* = 0.0067, followed by Tukey’s post hoc test; Fig. 1f) when compared with saline-injected control rats (Fig. 1c and f). Electrode localization in the STN was confirmed with Nissl staining, as represented in Fig. 1b. Eight animals had electrodes incorrectly positioned and were excluded from the study (*data not shown*). The electrode implantation per se (6-OHDA + DBS OFF group) did not prevent either 6-OHDA-induced loss of TH-IR (Fig. 1e) or asymmetric rotational behavior (Fig. 1f). As for the immobility test, striatal 6-OHDA increased the latency spent in the bar when compared to the saline group (Fig. 1g). The presence of the electrode itself (6-OHDA + DBS OFF group) significantly decreased the time spent in the bar when compared with the 6-OHDA group (1-w-ANOVA; *F*_(3,31)_ = 142.3, *p* < 0.0001, followed by Tukey’s post hoc test; Fig. 1g). STN-DBS reduced neurotoxin-induced behavioral immobility when compared to the 6-OHDA group (Fig. 1g) and baseline responses before DBS (Fig. 1h).

## STN-DBS Does not Alter the Hyperplasia of Astrocytes, but Changes the Inflammatory Pattern in the Globus Pallidus

**Fig. 2:**
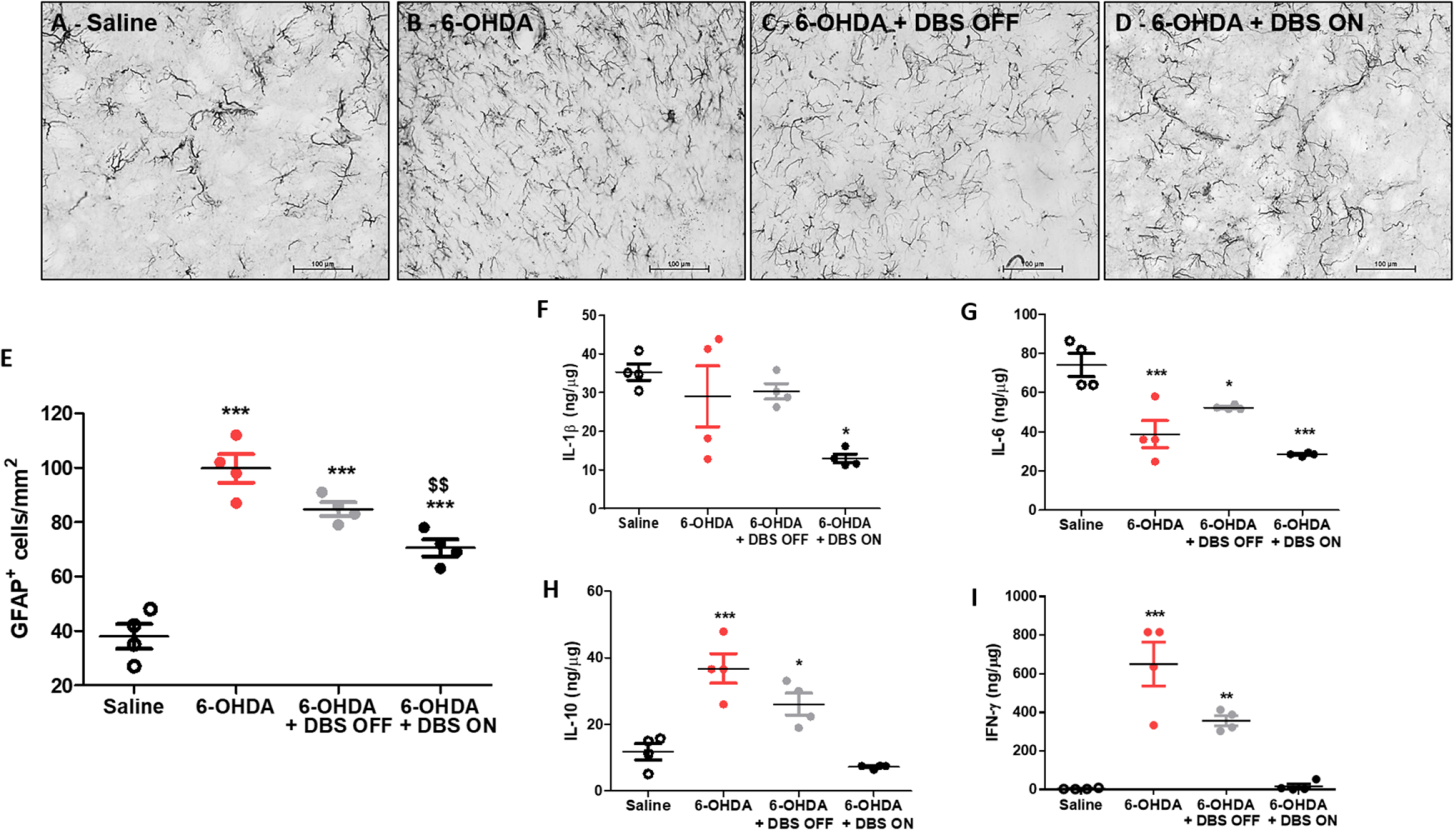
Pallidal inflammation following STN–DBS in vivo. **a**–**d** Photomicrographs of immunoreactivity for GFAP in saline (**a**), 6-OHDA (**b**), 6-OHDA + DBS OFF, (**c**) and 6-OHDA + DBS ON (**d**) and** e** quantification of GFAP + cells in the GP (*n* = 4). **f**–**i** In vivo evaluation of expression levels of IL-1β (**f**), IL-6 (**g**), IL-10, (**h**) and IFN-γ (**i**) in the GP. Values represent the mean + SEM of four independent experiments. *p < 0.05; **p < 0.001, ***p < 0.0001 vs. saline-injected control animals. $$*p* < 0.001 vs. 6-OHDA group

Thirteen days following its administration, 6-OHDA induced hyperplasia of astrocytes in the GP (1-w-ANOVA; *F*_(3,15)_ = 43.86, *p* < 0.0001, followed by Tukey’s post hoc test; Fig. 2b and e), when compared to saline-injected controls (Fig. 2a and e). In addition, while no difference in IL-1β expression was observed in the GP (1-w-ANOVA; *F*_(3,15)_ = 5.518, *p* > 0.05, followed by Tukey’s post hoc test; Fig. 2f), 6-OHDA induced a significant decrease in IL-6 protein levels (1-w-ANOVA; *F*_(3,15)_ = 18.54, *p* < 0.0001, followed by Tukey’s post hoc test; Fig. 2g) and an increase in IL-10 (1-w-ANOVA; *F*_(3,15)_ = 19.97, *p* < 0.0001, Fig. 2h) and IFN-γ expression (1-w-ANOVA; *F*_(3,15)_ = 27.60, *p* < 0.0001, followed by Tukey’s post hoc test; Fig. 2i) when compared to saline-injected control animals. DBS OFF was not able to attenuate 6-OHDA-induced astrocytic hyperplasia (Fig. 2a–d) in the GP, when compared to saline-injected control animals. In addition, DBS (6-OHDA + DBS ON group) significantly inhibited the effect of 6-OHDA on the hyperplasia phenomenon of astrocytes (Fig. 2e). Regarding cytokine levels, DBS-stimulated 6-OHDA animals presented decreased IL-1β expression, when compared to saline control animals (Fig. 2f), and completely attenuated IL-10 and IFN-γ expression (Fig. 2h and i, respectively), when compared to 6-OHDA-injected animals.

## TNF-α Induces Classical Inflammatory Activation of Astrocytes In Vitro

**Fig. 3:**
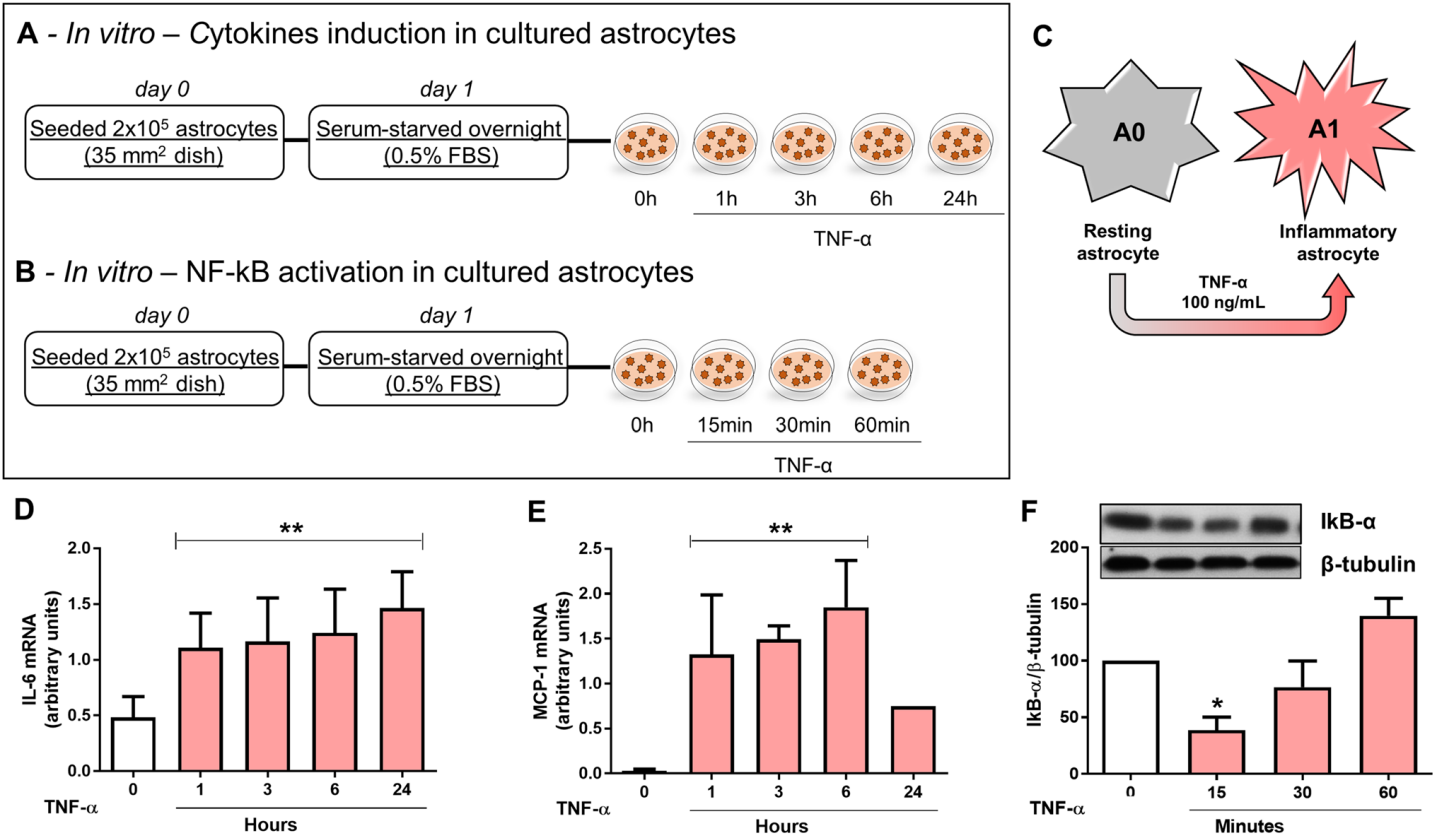
Effect of TNF-α on astrocyte activation in vitro. **a**, **b** In vitro experimental design and **c**, scheme representing astrocyte phenotypic switch from resting A0 to classical inflammatory A1. **d**, **e** Astrocytes were stimulated with TNF-α for 1, 3, 6, or 24 h and IL-6 (**d**) and MCP-1 (**e**) mRNA levels were analyzed by RT-PCR. Bar graphs represent means ± SD of five independent experiments normalized to RPL. **p < 0.001 when compared to control non-stimulated cells. **f** Astrocytes were stimulated with TNF-α (0, 15, 30 and 60 min) and IκB-α protein expression was evaluated by *western blotting* and normalized to β-tubulin. Bar graphs represent means ± SD of five independent experiments. *p < 0.05 vs. control non-stimulated cells

To determine whether cultured resting astrocytes (A0 subtype) would exhibit characteristics of classical inflammatory astrocytes (A1-like subtype) (Fig. 3c), cells were treated with TNF-α at different time points (1, 3, 6, and 24 h) and the expression of IL-6 and MCP-1 mRNA was analyzed. As expected, IL-6 mRNA levels (1-w-ANOVA; *F*_(4,19)_ = 4.925, *p* = 0.0097; followed by Tukey’s post hoc test; Fig. 3d) and MCP-1 levels (1-w-ANOVA; *F*_(4,19)_ = 10.46, *p* = 0.0072; followed by Tukey’s post hoc test; Fig. 3e) were found to be significantly upregulated following TNF-α treatment when compared with non-stimulated control cells. To investigate the time course of NF-κB activation in astrocytes in vitro, IκB-α degradation was monitored following TNF-α treatment for 15, 30, and 60 min. IκB-α protein levels were found to be significantly decreased 15 min after the TNF-α treatment and returned to baseline levels 60 min after stimulation, when compared with control non-stimulated cells (1-w-ANOVA; *F*_(4,19)_ = 3.68, *p* = 0.0171, followed by Tukey’s post hoc test; Fig. 3f).

## HFS Prevents MCP-1, but not IL-6 Induction in Cultured Astrocytes Following TNF-α Treatment

**Fig. 4:**
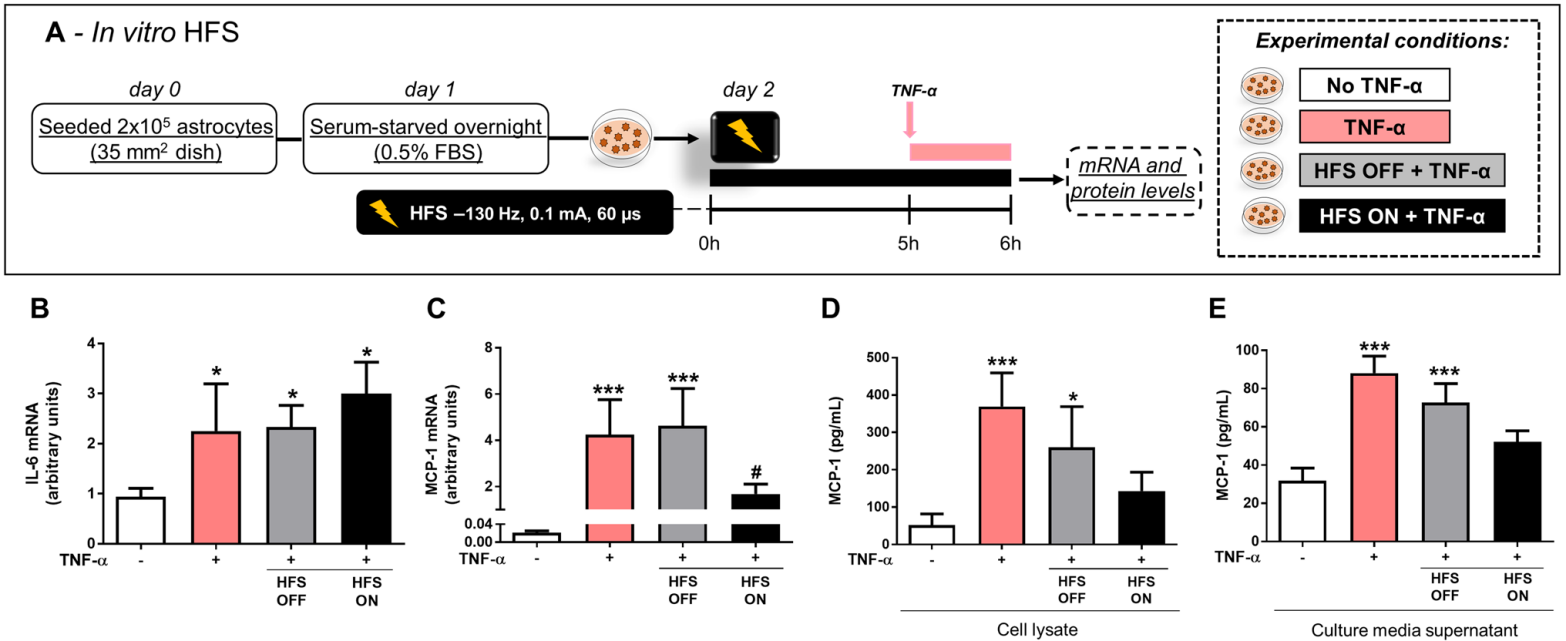
Effect of HFS on TNF-α-stimulated cytokine induction. **a** In vitro experimental design. Astrocytes were stimulated with HFS (HFS ON) for 6 h, TNF-α was added to the culture media during the last hour of stimulation, and cytokine mRNAs and proteins were measured. **b**, ** c** IL-6 (**b**) and MCP-1 mRNA expression (**c**) were analyzed by RT-PCR and normalized to RPL. Bar graphs represent means ± SD of five independent experiments. *p < 0.05, ***p < 0.001 vs. control non-stimulated cells and #*p* < 0.05 vs. TNF-α-treated cells. **d**, **e** MCP-1 protein expression was measured by ELISA in cell lysates (**d**) and in cell culture supernatant (**e**). Bar graphs represent means ± SD of five independent experiments. *p < 0.05 and ***p < 0.001 vs. control non-stimulated cells

To investigate whether HFS inhibits TNF-α-induced cytokine expression, astrocytes were exposed to 6 h of HFS and TNF-α was added to the culture media during the last hour of HFS stimulation (see experimental design in Fig. 4a). Similar to our in vivo findings (Fig. 2g), HFS ON did not prevent TNF-α-stimulated IL-6 mRNA levels induction, as shown in Fig. 4b (1-w-ANOVA; *F*_(3,19)_ = 3.994, *p* = 0.1071; followed by Tukey’s post hoc test). On the other hand, HFS ON prevented TNF-α-induced increases in MCP-1 mRNA levels (*F*_(3,19)_ = 14.96, *p* = 0.0002; followed by Tukey’s post hoc test; Fig. 4c). Corroborating our mRNA expression data, HFS ON prevented TNF-α-induced MCP-1 protein levels in cell lysates (1-w-ANOVA; *F*_(3,19)_ = 12.93, *p* = 0.0002; followed by Tukey’s post hoc test; Fig. 4d) and in the culture supernatant (*F*_(3,19)_ = 35.09, *p* < 0.0001; Fig. 4e). The presence of the electrode itself (TNF-α + HFS OFF group) did not prevent TNF-α-stimulated cytokine induction (TNF-α vs. TNF-α + HFS OFF group) (Fig. 4b–e).

## HFS Inhibits NF-κB Signaling Pathway in Astrocytes

**Fig. 5:**
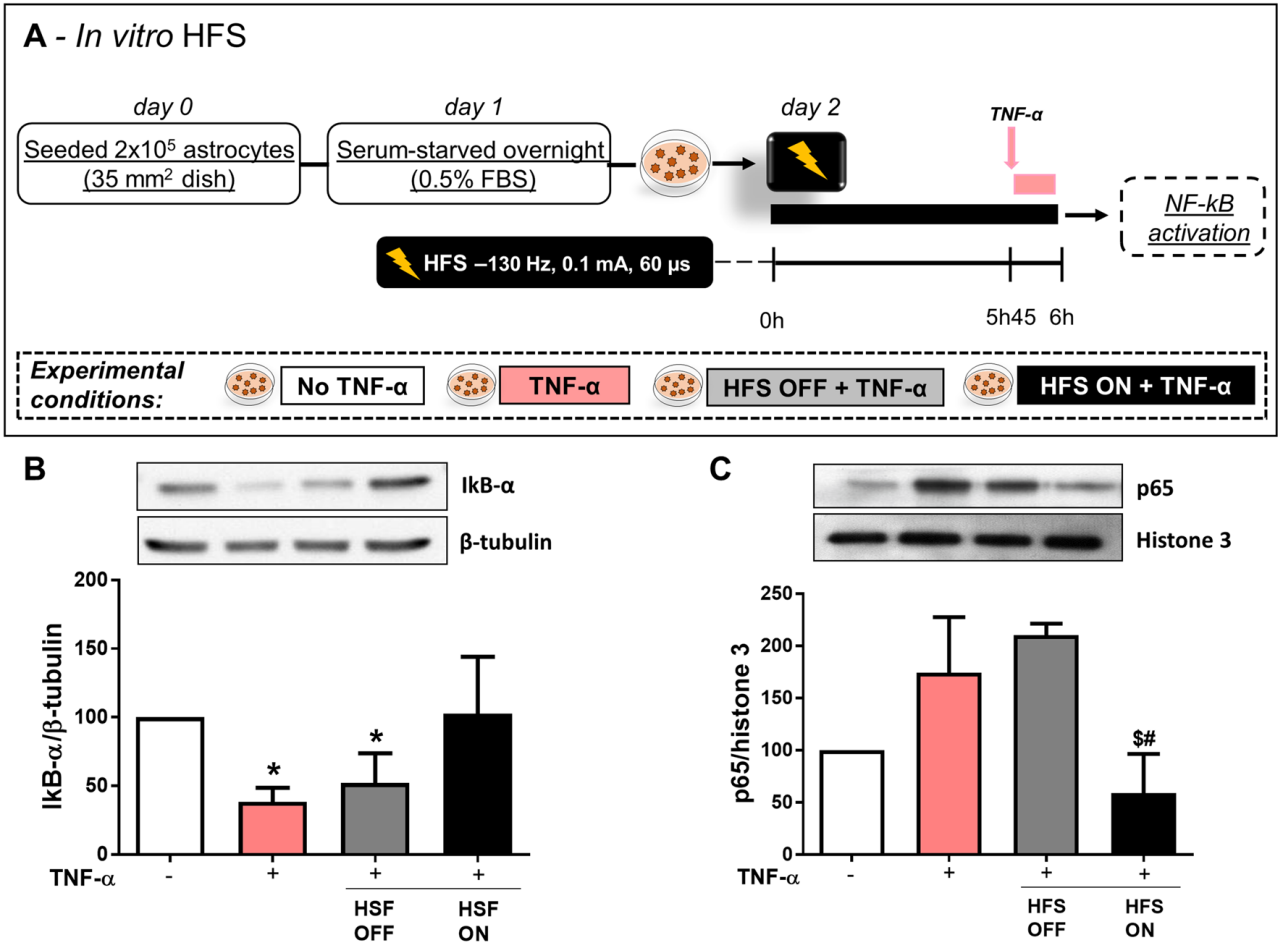
Effect of HFS on the NF-κB signaling pathway. **a**, **b** NF-κB signaling pathway was assessed by Western blot in astrocytes submitted to HFS (HFS ON, 6 h) and TNF-α (15 min) (**a**), and the IκB-α protein expression was evaluated in whole cell lysates using Western blotting and corrected for β-tubulin (**b**). **c** Astrocytes were stimulated with HFS and the p65 protein expression was evaluated in the nuclear fraction using Western blotting and corrected according to histone 3. Bar graphs represent means ± SD of five independent experiments. *p < 0.05 when compared to control no TNF-α; #*p* < 0.05 when compared to TNF-α + ; ^$^*p* < 0.05 when compared to TNF-α + HFS OFF

Because NF-κB is known to regulate transcription of MCP-1 (Rovin et al. 1995), we hypothesized that HFS affects the NF-κB signaling pathway. To investigate whether HFS interferes with the TNF-α-induced NF-κB activation in cultured astrocytes, the protein expression of the NF-κB inhibitor IκB-α was evaluated. Because IκB-α protein expression was found to be significantly decreased 15 min after TNF-α stimulation (Fig. 3f), we exposed astrocytes to HFS for 6 h, added TNF-α to their culture media during the last 15 min of stimulation (see experimental design in Fig. 5a), and evaluated IκB-α degradation. HFS significantly prevented IκB-α protein degradation (1-w-ANOVA; *F*_(3,19)_ = 9.050, *p* = 0.0012; followed by Tukey’s post hoc test; Fig. 5b). In addition, HFS also prevented TNF-α-induced p65 translocation to the nuclear fraction in cultured astrocytes (1-w-ANOVA; *F*_(3,19)_ = 12.45, *p* = 0.0015; followed by Tukey’s post hoc test; Fig. 5c).

## Discussion

STN–DBS has been widely used for the treatment of advanced PD when long-term treatment with dopaminergic agents induces debilitating side effects (Kumar et al. 1998; Benabid et al. 2009; Cury et al. 2016; dos-Santos-Pereira et al. 2016). DBS at intermediate stages of PD has been shown to provide superior benefits when compared to advanced PD, preventing severe complications resulting from medication at high doses, tolerability, and optimization of the technique’s effectiveness. Early DBS has been shown to improve quality of life and delay the appearance of motor fluctuations, dyskinesias, and functional disability (Charles et al. 2012; Hacker et al. 2015, 2016). Considering all the aforementioned observations, we have chosen to use a DBS protocol at the early-onset of 6-OHDA-induced PD, in which approximately 50% of dopaminergic neuronal loss is observed in the SN 7 days after a 6-OHDA injection, mimicking the neuronal loss observed in patients at earlier stages of PD (Marsden 1990; Lang and Lozano 1998; Dauer and Przedborski 2003; Ross et al. 2004).

We show that STN–DBS was able to improve motor impairment (akinesia) induced by 6-OHDA. In addition, our data is consistent with clinical studies in which STN–DBS significantly enhanced daily activities and improved motor symptoms in the “off” medication condition (Kleiner-Fisman et al. 2006). Remarkably, it has been observed that the electrode insertion effect in the STN per se seems to effectively improve PD symptoms (Benazzouz et al. 1993; Benabid et al. 1994), probably by mimicking the effects of STN inactivation (Florence et al. 2016; Hamani et al. 2017). In our study, we observed a 25% reduction in the time animals spent in the bar in the 6-OHDA + DBS OFF group when compared to 6-OHDA animals with no electrode implanted. Because of the small dimensions of the STN, this response could have occurred due to a STN microlesion effect (Fig. 1b). A limitation of our in vivo experimental approach is that the ratio of electrode size to target in rodents is very different from that observed in humans. We note, however, that microlesion effects are also commonly observed in clinical practice.

Several PD models in rats have been shown to induce glial cell activation in the SN and striatum (McGeer and McGeer 2008; Sanchez-Guajardo et al. 2015; Booth et al. 2017; Joers et al. 2017). GFAP staining has been widely used as a marker of reactive astrocytes. Its increased expression is characteristic of astrocytic hyperplasia/hypertrophy (Sofroniew and Vinters 2010) which is a hallmark of neuropathological conditions such as Alzheimer’s and PD (McGeer and McGeer 2008). Reactive gliosis around implanted DBS electrodes has been reported (Stock et al. 1979; Haberler et al. 2000; Sun et al. 2008). In astrocytes, electrical stimulation induces hyperpolarization and low input resistance compared to neurons (Amzica and Neckelmann 1999; Amzica et al. 2002; Mishima et al. 2007). As a consequence, HFS/DBS induces an increase in adenosine and glutamate levels in astrocytes and seems to induce astrocytic Ca^2+^ waves that propagate away from the stimulation site (Bekar et al. 2008; Tawfik et al. 2010). Additional studies have proposed that HFS may protect the brain by regulating astrocytes (Fenoy et al. 2014). Recently, Jang and collaborators demonstrated that HFS induces the release of extracellular matrix proteins, such as insulin growth factor (IGF)-1 pathway, from human astrocytes in vitro (Jang et al. 2019), which protects neurons from excitotoxicity (Chen et al. 2019). However, the HFS/DBS effect on inflammatory astrocytes and its ability to affect the astrocytic secretory phenotype is completely unknown. Our in vivo data obtained from GP immunostained for GFAP demonstrates that STN–DBS partially reverses the effect of 6-OHDA on the hyperplasia phenomenon, which suggests an inhibition of astrocytic activation by DBS. It is important to note that astrocytic hyperplasia and increases in GFAP expression have also been described in conditions where astrocytes are believed to play an anti-inflammatory neuroprotective role (alternative A2 phenotype) (Barreto et al. 2011; Liddelow et al. 2017). Since DBS does not prevent 6-OHDA-induced astrocytic hyperplasia in the GP, future investigation should focus on whether DBS promotes astrocytic alternative anti-inflammatory (A2) activation.

Reactive astrogliosis is associated with increased release of pro-inflammatory cytokines such as TNF-α and IFN-γ, promoting inflammatory activation of microglial cells that further contributes to synaptic damage and neurodegeneration (McGeer and McGeer 2011). Several inflammatory cytokines including TNF-α, IFN-γ, and MCP-1 have been described in brain tissue, spinal fluid, and peripheral blood of PD patients (Gao and Hong 2008; Reale et al. 2009; Whitton 2009; Banks and Erickson 2010; Collins et al. 2012). We demonstrate that 6-OHDA not only induced an increase in the number of GFAP-positive cells in the GP 13 days after PD model induction, but also in IFN-γ induction. In addition, 6-OHDA-induced IFN-γ was completely attenuated by STN-DBS. IFN-γ is a pro-inflammatory cytokine that acts as a potent glial activator (Schroder et al. 2004) and polarizes M0 subtype (resting state) macrophages/microglia to M1 subtype (pro-inflammatory state) (Chistiakov et al. 2018). In animal studies, increases in this cytokine are associated with the loss of dopaminergic neurons, nigrostriatal degeneration, and motor impairment (Mount et al. 2007; Barcia et al. 2011; Chakrabarty et al. 2011). In PD patients, high levels of IFN-γ have been detected in the SN, striatum, cerebrospinal fluid, and plasma (Mogi et al. 1996; Hunot et al. 1999; Mount et al. 2007), which seems to contribute to the induction and maintenance of the neurodegenerative process (Gerhard et al. 2006; Brodacki et al. 2008). Our report is the first to show that the striatal 6-OHDA model produces pallidal IFN-γ induction and that STN-DBS can reverse this phenomenon. Consistent with our findings, STN-DBS decreased IFN-γ derived from T-helper-1 cells in PD, which seems to correlate with DBS efficacy (Soreq et al. 2013).

IL-10 is an anti-inflammatory cytokine released by glial cells in the central nervous system (CNS), and in response to 6-OHDA, it is also produced by T-helper-2 cells (Soreq et al. 2013). In the present study, we found an increase in pallidal IL-10 levels in response to 6-OHDA, which was completely abolished by DBS. Since IL-10 was shown to be a potent IFN-γ inhibitor (Aharoni et al. 2000; Rengarajan et al. 2000), the increase in IL-10 levels following 6-OHDA may serve to inhibit or attenuate IFN-γ production in an attempt to control the inflammatory response. Hence, the decreased levels of IL-10 after DBS may be a consequence of stimulation-induced decrease of IFN-γ.

Previous studies have shown that STN–DBS was able to decrease IL-6 expression in epileptic rats (Amorim et al. 2015; Chen et al. 2017). We found that IL-6 protein levels are significantly downregulated in the GP following 6-OHDA, when compared to saline-injected control rats. No detectable differences in pallidal IL-6 levels were found between 6-OHDA and 6-OHDA + DBS ON groups. Other studies showed no difference in IL-6 protein levels in both SN and striatum 12 days after 6-OHDA-induced PD in rats (Koprich et al. 2008).

Our data demonstrates that DBS significantly decreases 6-OHDA-induced astrocytic hyperplasia in the GP. Because of the importance of astrocytes in modulating the synaptic environment, these cells have been considered key mediators of DBS efficacy (Fenoy et al. 2014). In addition, astrocytes are an important source of pro-inflammatory cytokines and play a pivotal role in brain inflammation (Sofroniew 2014). We therefore used a model of mouse cultured astrocytes exposed to HFS and TNF-α to mimic in vivo DBS in PD and test whether HFS regulates cytokine induction in astrocytes. While HFS does not seem to inhibit increased IL-6 mRNA levels, our data suggests that HFS significantly attenuates TNF-α-stimulated MCP-1 induction at both the protein and mRNA levels. MCP-1 is a potent monocyte-attracting chemokine and has also been shown to attract M1 microglia to inflammation sites (Hinojosa et al. 2011), contributing to further enhance local brain neuroinflammation (Deshmane et al. 2009). The fact that HFS failed to prevent TNF-α-induced IL-6 expression suggests that HFS specifically affects the secretion of determined cytokines and potentially specific transcription factors. While transcriptional regulation of IL-6 in astrocytes is mediated by both the pro-inflammatory transcription factor NF-κB and the transcription factor activated by tyrosine kinases STAT3 (Hirano et al. 2000), MCP-1 expression is mainly regulated by NF-κB (Rovin et al. 1995; Schwamborn et al. 2003; Widera et al. 2004; Kigerl et al. 2009). NF-κB is a major CNS transcription factor and plays a pivotal role in glial cell function (O’Neill and Kaltschmidt 1997). Cytokines such as TNF-α activate the NF-κB signaling pathway by inducing the degradation of IκB-α, an NF-κB cytoplasmic inhibitor protein (Baeuerle and Baltimore 1988). Once IκB-α is degraded, the NF-κB p65 subunit translocates into the nuclei, activating the transcription of inflammatory cytokines (Baldwin 1996). In this study, we report that HFS not only regulates cytokine induction, but is also a novel regulator of NF-κB activation in astrocytes. Our results indicate that HFS prevents IκB-α degradation and inhibits TNF-α-induced p65 nuclear translocation, supporting an uncovered anti-inflammatory role for HFS in astrocytes. A limitation of our in vitro study is that experiments were performed in an astrocyte mouse cell line rather than a rat cell line. However, the concordance between in vitro and in vivo data makes this unlikely to be an issue.

**Fig. 6:**
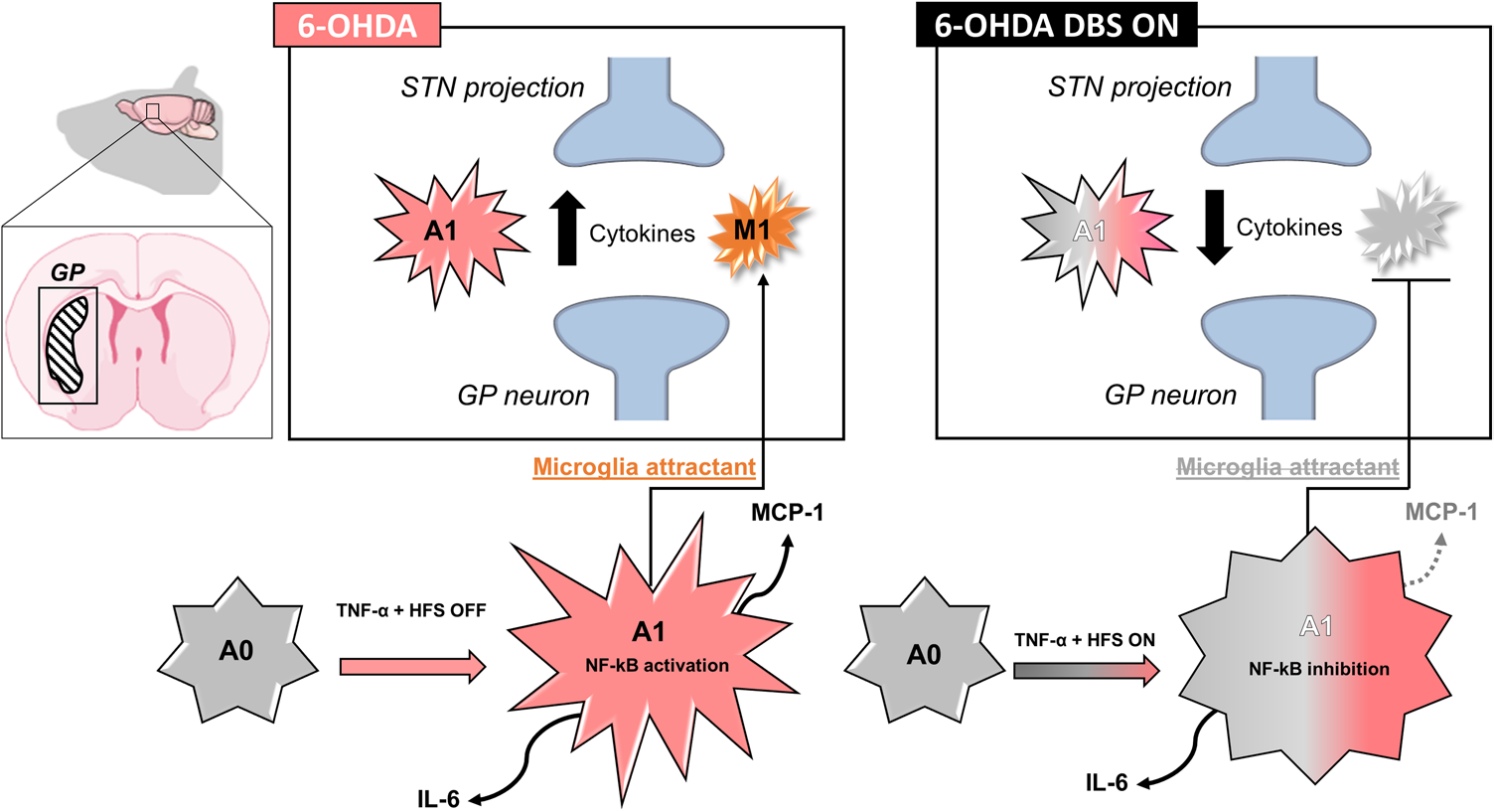
Representative DBS/HFS scheme suggested mechanism. Six-OHDA induces inflammation within the GP by activating A1, which attracts microglia cells and increases cytokines. STN–DBS/HFS while only slightly changing the number of astrocytes attenuates the classical inflammatory A1 astrocyte subtype by inhibiting NF-κB activation and cytokine release. The less activated astrocytes decrease microglia attraction reflecting in the inhibition of local cytokine release leading to improvement of inflammation within the GP

We propose that one of the mechanisms by which DBS/HFS inhibits neuroinflammation and improves motor impairment following PD is by decreasing astrocyte A1 subtype activation and microglia attractant, hence attenuating the overall inflammatory process (Fig. 6). Our findings demonstrate for the first time that DBS/HFS has a crucial role in inflammation inhibition, potentially regulating NF-κB activation. We suggest that DBS/HFS inhibits classical astrocytic activation, decreasing overall inflammation, while improving PD symptoms.

## Abbreviations

6-OHDA: 6-Hydroxydopamine
DBS: Deep brain stimulation
FBS: Fetal bovine serum
GFAP: Glial fibrillary acidic protein
GP: Globus pallidus
HFS: High-frequency stimulation
IκB: Nuclear factor of kappa light polypeptide gene enhancer in B-cell inhibitor
IFN: Interferon
IL: Interleukin
iNOS: Inducible nitric oxide synthase
IR: Immunoreactivity
MCP: Monocyte chemoattractant protein
NF-κB: Nuclear factor kappa-light-chain-enhancer of activated B cells
PB: Phosphate buffer
PD: Parkinson’s disease
PFA: Parafolmaldehyde
SN: Substancia nigra
STN: Subthalamic nucleus
TH: Tyrosine hydroxylase
TNF: Tumor necrosis factor
